# MicroRNA-124 as a novel treatment for persistent hyperalgesia

**DOI:** 10.1186/1742-2094-9-143

**Published:** 2012-06-25

**Authors:** Hanneke LDM Willemen, Xiao-Jiao Huo, Qi-Liang Mao-Ying, Jitske Zijlstra, Cobi J Heijnen, Annemieke Kavelaars

**Affiliations:** 1 Laboratory of Neuroimmunology and Developmental Origins of Disease (NIDOD), University Medical Center Utrecht, Utrecht, 3584 EA, The Netherlands; 2 Integrative Immunology and Behavior Program, College of ACES and College of Medicine, University of Illinois at Urbana-Champaign, Urbana, IL, 61801, USA

**Keywords:** miR-124, Microglia/macrophages, Inflammatory hyperalgesia, Neuropathic pain, GRK2, M1/M2 balance

## Abstract

**Background:**

Chronic pain is often associated with microglia activation in the spinal cord. We recently showed that microglial levels of the kinase G protein–coupled receptor kinase (GRK)2 are reduced in models of chronic pain. We also found that mice with a cell-specific reduction of around 50% in GRK2 level in microglia/macrophages (LysM-GRK2^+/−^ mice) develop prolonged inflammatory hyperalgesia concomitantly with ongoing spinal microglia/macrophage activation. The microRNA miR-124 is thought to keep microglia/macrophages in brain and spinal cord in a quiescent state. In the present study, we investigated the contribution of miR-124 to regulation of hyperalgesia and microglia/macrophage activation in GRK2-deficient mice. In addition, we investigated the effect of miR-124 on chronic inflammatory and neuropathic pain in wild-type (WT) mice.

**Methods:**

Hyperalgesia was induced by intraplantar IL-1β in WT and LysM-GRK2^+/−^ mice. We determined spinal cord microglia/macrophage miR-124 expression and levels of pro-inflammatory M1 and anti-inflammatory M2 activation markers. The effect of intrathecal miR-124 treatment on IL-1β-induced hyperalgesia and spinal M1/M2 phenotype, and on carrageenan-induced and spared nerve injury-induced chronic hyperalgesia in WT mice was analyzed.

**Results:**

Transition from acute to persistent hyperalgesia in LysM-GRK2^+/−^ mice is associated with reduced spinal cord microglia miR-124 levels. In our LysM-GRK2^+/−^ mice, there was a switch towards a pro-inflammatory M1 phenotype together with increased pro-inflammatory cytokine production. Intrathecal administration of miR-124 completely prevented the transition to persistent pain in response to IL-1β in LysM-GRK2^+/−^ mice. The miR-124 treatment also normalized expression of spinal M1/M2 markers of LysM-GRK2^+/−^ mice. Moreover, intrathecal miR-124 treatment reversed the persistent hyperalgesia induced by carrageenan in WT mice and prevented development of mechanical allodynia in the spared nerve injury model of chronic neuropathic pain in WT mice.

**Conclusions:**

We present the first evidence that intrathecal miR-124 treatment can be used to prevent and treat persistent inflammatory and neuropathic pain. In addition, we show for the first time that persistent hyperalgesia in GRK2-deficient mice is associated with an increased ratio of M1/M2 type markers in spinal cord microglia/macrophages, which is restored by miR-124 treatment. We propose that intrathecal miR-124 treatment might be a powerful novel treatment for pathological chronic pain with persistent microglia activation.

## Background

Micro (mi) RNAs regulate expression of multiple genes by promoting degradation of mRNA or by preventing translation of target genes. It has recently been reported that high levels of the miRNA miR-124 are present in resident microglia in brain and spinal cord. Ponomarev *et al*. showed that microglia activated *in vitro* and *in vivo* have low levels of miR-124. In addition, peripheral macrophages, which have an activated phenotype, express low levels of miR-124 compared with isolated naive microglia from brain and spinal cord. Based on such findings, it has been suggested that high levels of miR-124 are required to keep microglia in a quiescent state [1].

Microglia/macrophages in the spinal cord play a key role in the development of chronic pain in rodent models of neuropathic pain, diabetic neuropathy, and chronic inflammatory pain [2-7]. However, it is not known whether microglial miR-124 contributes to chronic pain.

Recently, we reported that chronic inflammatory hyperalgesia and neuropathic pain are associated with decreased levels of the serine–threonine kinase G-protein receptor kinase (GRK)2 in spinal microglia/macrophages [8,9]. GRK2 regulates cellular signaling by promoting desensitization of agonist-occupied G protein-coupled receptors and by direct interaction with multiple downstream signaling pathways [10-14]. Our recent functional studies indicated a key role for GRK2 in regulating the transition from acute to persistent hyperalgesia. In mice with a 50% reduction of GRK2 in microglia/macrophages (LysM-GRK2^+/−^ mice), thermal and mechanical hyperalgesia induced by intraplantar injection of inflammatory mediators were markedly prolonged [8-10,15]. For example, thermal hyperalgesia induced by a single intraplantar injection of the pro-inflammatory cytokine IL-1β resolved within 24 hours in wild-type (WT) mice, but lasted for at least 8 days in LysM-GRK2^+/−^ mice. Similarly, low-dose carrageenan-induced hyperalgesia was prolonged from 3 to 4 days in WT mice to 20 days in LysM-GRK2^+/−^ mice. This prolongation of hyperalgesia in LysM-GRK2^+/−^ mice was reversible by intrathecal administration of the microglial/macrophage inhibitor minocycline, indicating a decisive role for spinal cord microglia/macrophage activity in regulating the transition to persistent hyperalgesia in these models [8,9].

It is now well known that macrophage activation can induce development of two functional subtypes known as pro-inflammatory (M1-type) and anti-inflammatory (M2-type) macrophages. These two activated types of macrophages can be discriminated by the expression of specific markers on their cell surface, and are characterized by expression of pro-inflammatory factors such as interleukin 1β, and inducible nitrous oxide synthase (iNOS) (in M1 macrophages) or anti-inflammatory cytokines including transforming growth factor (TGF)-β (in M2 macrophages). In addition, there is evidence that microglial activation can also lead to development of either the M1 or M2 phenotype [16,17]. However, it is not known whether or how these distinct macrophages/microglia phenotypes contribute to regulation of pain responses.

In this study we investigated whether low levels of microglial/macrophage GRK2 promote the transition to chronic hyperalgesia via a miR-124-mediated pathway in spinal microglia/macrophages, and whether low GRK2 is associated with the expression of the M1 and M2 phenotype in spinal-cord microglia. We also determined whether miR-124 can be used to treat persistent hyperalgesia in models of chronic inflammatory and neuropathic pain in WT mice.

## Methods

Experiments were performed in accordance with international guidelines and approved by the experimental animal committee of UMC Utrecht.

### Animals

We used female mice (aged 12 to 14 weeks) with cell-specific reduction of GRK2 in microglia/macrophages (LysM-GRK2^+/−^), and control LysM-GRK2^+/+^ mice [8,9] (WT) littermates bred and maintained in the animal facility of the University of Utrecht, The Netherlands). LysM-Cre mice and GRK2-fLox mice were obtained from Jackson Laboratories.

Mice received an intraplantar injection in the hind paw of 5 μl recombinant murine IL-1β (200 ng/ml; Peprotech, Rocky Hill, NJ, USA) or 20 μl λ-carrageenan (2% w/v; Sigma-Aldrich, St. Louis, MO, USA) diluted in saline. Heat-withdrawal latency times were determined using the Hargreaves test (IITC Life Science, Woodland Hills, CA) as described previously [18]. Intraplantar injection of saline did not induce detectable hyperalgesia in any of the genotypes.

Spared nerve injury (SNI) was performed in male C57/bl6 mice as described previously [19]. Briefly, under 2% isoflurane anesthesia, the sciatic nerve and its three terminal branches were exposed carefully with minimal damage. Leaving the sural nerve intact, the common peroneal and the tibial nerves were tightly ligated with 6–0 silk surgical sutures and cut 2 to 4 mm distal to the ligation. Sham controls underwent anesthesia, incision, and exposure of the nerve only.

Mechanical allodynia was measured using von Frey hairs as described previously [9]. The 50% paw-withdrawal threshold was calculated using the up-and-down method [20]. In brief, animals were placed on a wire-grid base, through which the von Frey hairs (Stoelting, Wood Dale, IL, USA) were applied (bending force range from 0.02 to 1.4 g, starting with 0.16 g). The hair force was increased or decreased depending on the response. Clear paw withdrawal, shaking, or licking were considered as nociceptive-like responses. Ambulation was considered an ambiguous response, and in such cases, the stimulus was repeated.

Spontaneous locomotor activity (LMA) was determined 3 days after SNI in a clean, novel cage similar to the home cage, but devoid of bedding or litter. The cage was divided into four virtual quadrants, and LMA was measured by counting the number of line crossings over a five-min period.

All behavioral experiments were performed by an experimenter blinded to treatment and in a randomized fasion.

The miRNA-124 (2 μg in 50 μl PBS, PM10691Applied Biosystems, Carlsbad, CA, USA) or control miRNA (2 μg in 50 μl PBS ,AM17110; Applied Biosystems) was mixed with transfection reagent (Lipofectamine 2000; Invitrogen, Paisley, UK) , diluted to the appropriate concentration (3 μl in 50 μl PBS) and applied intrathecally (5 μl/mouse) while the animals were under light isoflurane anesthesia.

### Spinal microglia and peripheral macrophage isolation

At 24 hours after intraplantar IL-1β injection, lumbar enlargements (L2-L5) or thoracic (T6-T10) spinal cord of four mice were pooled and the microglia were isolated by Percoll density gradient centrifugation as described previously [9].

Peripheral macrophages were isolated from peritoneal lavages using CD11b magnetic beads, in accordance with the manufacturer’s instructions (BD IMag, San Diego, CA, USA).

### MicroRNA-124 and mRNA expression analysis

Total RNA was isolated using a commercial kit (RNeasy Mini Kit; Qiagen, Hilden, Germany). Specific reverse transcriptase and Taqman miRNA assays (Applied Biosystems) were carried out in accordance with the manufacturer’s instructions. The miR-124 expression (mmu-miR-124a, 001182) was normalized for snoRNA55 (001228) expression.

### Real-time reverse transcriptase PCR

cDNA was processed from total RNA using reverse transcriptase (SuperScript; Invitrogen), and real-time PCR was performed (iQ5 Real-Time PCR Detection System; Bio-Rad, Alphen a/d Rijn, The Netherlands) using the primers shown in Table 1. Data were normalized for GAPDH and actin expression.

**Table 1:** Primers used for real-time reverse transcriptase PCR.

Name	Direction	Primer sequence 5′ → 3′
C/EBP-α	Forward	AgCTTACAACAggCCAggTTTC
	Reverse	CggCTggCgACATACAgTAC
TGF-β	Forward	CAgAgCTgCgCTTgCAgAg
	Reverse	gTCAgCAgCCggTTACCAAg
iNOS	Forward	ACCCACATCTggCAgAATgAg
	Reverse	AgCCATgACCTTTCgCATTAg
IL-1β	Forward	CAACCAACAAgTgATATTCTCCATg
	Reverse	gATCCACACTCTCCAgCTgCA
GAPDH	Forward	TgAAgCAggCATCTgAggg
	Reverse	CgAAggTggAAgAgTgggAg
Actin	Forward	AgAgggAAATCgTgCgTgAC
	Reverse	CAATAgTgATgACCTggCCgT

Abbreviations: *C/EBP* CCAAT-enhancer-binding protein, *GAPDH* glyceraldehyde 3-phosphate dehydrogenase, *IL* interleukin, *iNOS* inducible nitrous oxide synthase, *TGF* transforming growth factor.

### Immunohistochemistry

Mice were perfused intracardially with PBS 15 hours after intraplantar IL-1β injection, followed by 4% paraformaldehyde in PBS. Spinal cords were post-fixed in 4% paraformaldehyde for 6 hours at 4 °C, and cryoprotected in sucrose. Cryosections (10 μm) of thoracic segments T6 to T10 and lumbar segments L2 to L5 were incubated with 1:100 goat anti-mouse CD206 (R&D Systems, Minneapolis, MN, USA), 1:200 goat anti-mouse aginase I (Santa Cruz Biotechnology, Santa Cruz, CA, USA) or 1:500 rat anti-mouse CD16/32 (BD Biosciences) followed by alexa flour 488-conjugated secondary antibodies. Sections were viewed under a fluorescence microscope (Axio Observer; Zeiss, Jena, Germany). M1 and M2 expression was quantified in approximately 10 to 15 dorsal horns of spinal cords per group (four mice per group). The level of expression in the lumbar or thoracic part from control WT mice was set at 100%.

### Statistical analysis

All data are presented as mean ± SEM. Measurements were compared using Student’s *t*-test or two-way ANOVA with Bonferroni post-hoc tests using Prism 4 software.

## Results

### MicroRNA-124 expression in spinal cord microglia and in peripheral macrophages

**Figure 1:**
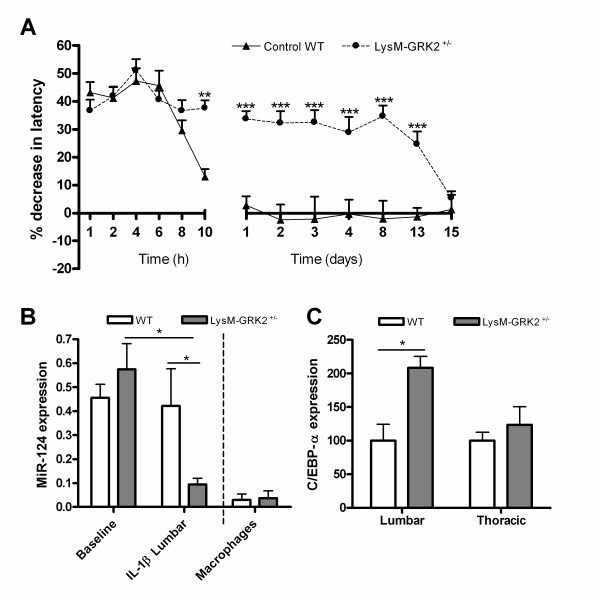
**MicroRNA-124 and CCAAT-enhancer-binding protein (C/EBP)-α expression after intraplantar interleukin (IL)-1β in wild-type (WT) and LysM-G protein–coupled receptor kinase (GRK)2**^**+/−**^**mice.** Control WT and LysM-GRK2^+/−^ mice received an intraplantar injection of 1 ng IL-1β. (**A**) Percentage decrease in heat withdrawal latency after the injection in control WT and LysM-GRK2^+/−^ mice (n = 12). At baseline (n = 9) and 24 hours (n = 6) after IL-1β injection, lumbar (L2 to L5) spinal cord microglia were isolated to determine (**B**) miR-124 expression (normalized for small nucleolar (sno)RNA55) and (**C**) the lumbar and thoracic mRNA expression of C/EBP-α normalized for glyceraldehyde-3-phosphate dehydrogenase (GAPDH) and actin) in control WT (n = 6) and LysM-GRK2^+/−^ (n = 6) mice. n = One sample contains isolated microglia from four mice. Data are expressed as means ± SEM. *P < 0.05, **P < 0.01, ***P < 0.001.

First, we examined whether the transition from acute IL-1β-induced hyperalgesia to persistent hyperalgesia in LysM-GRK2^+/−^ mice is associated with changes in the level of miR-124 in spinal cord microglia. We isolated lumbar spinal cord microglia at baseline and 24 hours after intraplantar IL-1β injection from WT and LysM-GRK2^+/−^ mice. At this time point, intraplantar IL-1β-induced hyperalgesia had resolved completely in the WT mice, whereas the LysM-GRK2^+/−^ mice were still hyperalgesic (Figure 1A). These data confirm our earlier results [9].

At 24 hours after intraplantar injection of IL-1β, the level of miR-124 in microglia isolated from the lumbar spinal cord of LysM-GRK2^+/−^ mice was significantly lower than that of spinal microglia from WT mice (Figure 1B). At baseline (without stimulus), no difference was seen between WT and LysM-GRK2^+/−^ mice in miR-124 expression in microglia from spinal cord or in macrophages from the peritoneal cavity (Figure 1B). In line with data in the literature, the basal expression of miR-124 was lower in peripheral macrophages than in spinal cord microglia [1]. There was no significant difference in miR-124 mRNA between microglia isolated from thoracic spinal (T6 to T10) cord of WT and LysM-GRK2^+/−^ mice after intraplantar IL-1β (data not shown). Therefore, thoracic spinal cord was used as a control in subsequent experiments.

It is thought that miR-124 regulates microglia/macrophage activity by downregulating the expression of CCAAT-enhancer-binding protein (C/EBP)-α, a transcription factor regulating myeloid cell differentiation [1,21,22]. Consistent with this notion, we found a significant increase in C/EBP-α mRNA in microglia isolated from the lumbar spinal cord of LysM-GRK2^+/−^ mice compared with microglia from control WT mice after intraplantar IL-1β (Figure 1C). C/EBP-α expression was similar in microglia isolated from control thoracic spinal cord of LysM-GRK2^+/−^ and control WT mice injected intraplantarly with IL-1β (Figure 1C).

### Spinal cord M1/M2 markers in interleukin-1β-induced persistent hyperalgesia

**Figure 2:**
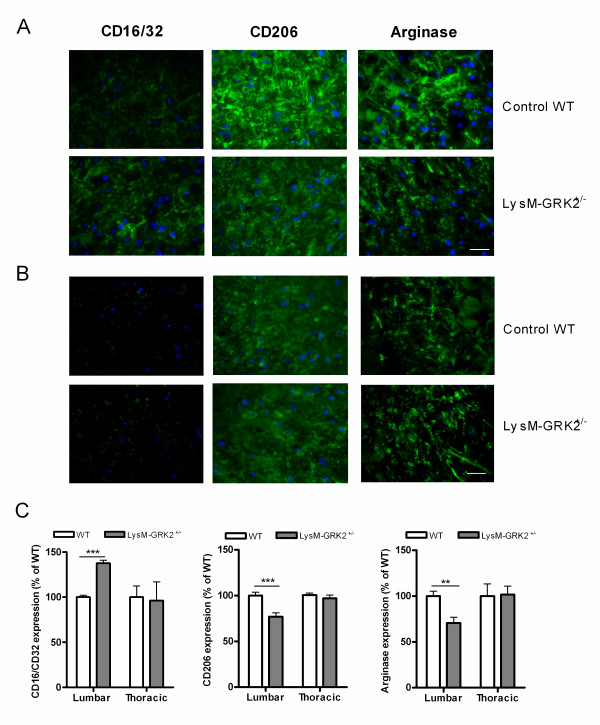
**M1 and M2 phenotype in spinal cord after intraplantar IL-1β.** Wild-type (WT) and LysM-G protein–coupled receptor kinase (GRK)2^+/−^ mice received an intraplantar injection of 1 ng IL-1β. At 15 hours after injection, spinal cord was collected, and frozen sections of (**A**) lumbar spinal cord (L2 to L5) and as control (**B**) thoracic spinal cord (T6 to T10) were stained for M1 (CD16/32) and M2 (CD206 and arginase-I) phenotypic markers. A representative example of M1 and M2 staining in the dorsal horn of one of the four mice per group is displayed. Scale bar indicates 20 μm. (**C**) Quantification of microglia/macrophages expressing M1 and M2 phenotypic markers in spinal cord from WT and LysM-GRK2^+/−^ mice. Expression was quantified in approximately 10 to 15 dorsal horns of spinal cords per group (4 mice per group). The level of expression in the lumbar or thoracic area from control WT mice was set at 100%. Data are expressed as means ± SEM. **P < 0.01, ***P < 0.001.

We next addressed the question of whether low GRK2 and decreased expression of miR-124 in spinal cord microglia are related to differences in the expression of microglia/macrophage activation markers in the spinal cord. We examined the expression of the markers of the classically activated (M1) microglia/macrophages CD16/32 and the alternatively activated (M2) cells arginase-I and CD206 by immunohistochemistry. After intraplantar injection of IL-1β, expression of the M1 marker CD16/32 in lumbar spinal cord was higher in LysM-GRK2^+/−^ mice than in WT mice. There were no differences in M1 marker expression between thoracic spinal cord of WT and LysM-GRK2^+/−^ (Figure 2A-C). Conversely, the level of expression of the M2 markers CD206 and arginase-I after intraplantar IL-1β injection was lower in lumbar spinal cord of LysM-GRK2^+/−^ mice compared with that of WT mice. Thoracic spinal cord of both genotypes did not differ in M2 marker expression (Figure 2A-C). In control thoracic spinal cord, no genotype-related differences were seen in the expression of M1 and M2 markers (Figure 2B,C).

**Figure 3:**
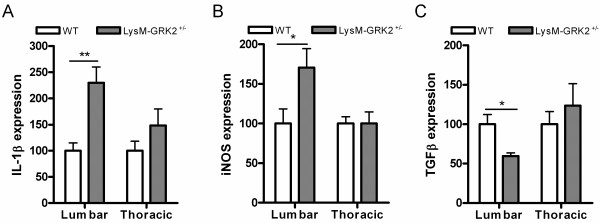
**Gene expression of M1-associated and M2-associated genes in spinal microglia after intraplantar injection of interleukin (IL)-1β.** Wild-type (WT) and LysM-G protein–coupled receptor kinase (GRK)2^+/−^ mice received an intraplantar injection of 1 ng IL-1β into the hind paws. At 24 hours after injection lumbar (L2 to L5) spinal cord and (as a control) thoracic (T6 to T10) spinal cord was collected from WT and LysM-GRK2^+/−^ mice, and microglia were isolated for analysis of mRNA expression by quantitative RT-PCR of **(A)** IL-1β, **(B)** inducible nitrous oxide synthase (iNOS) and **(C)** transforming growth factor (TGF)-β (n = 6 quantiatitve PCR samples per group; one sample contains isolated microglia from 4 mice). Data are expressed as means ± SEM. *P < 0.05, **P < 0.01.

As a functional correlate of the M1 and M2 phenotype, we quantified mRNA encoding the pro-inflammatory (M1) cytokine IL-1β, the M1-related enzyme iNOS, and the anti-inflammatory (M2) cytokine TGF-β [23-26]. We injected LysM-GRK2^+/−^ or WT mice with intraplantar IL-1β, and isolated microglia from lumbar spinal cord 24 hours later. Compared with control WT mice, freshly isolated microglia from LysM-GRK2^+/−^ mice contained significantly more mRNA for pro-inflammatory IL-1β and iNOS, and less mRNA for anti-inflammatory TGF-β,(Figure 3). As a control, we isolated microglia from the thoracic spinal cord of the same animals, and did not observe any difference in cytokine expression between genotypes in these cells (Figure 3).

### MicroRNA-124 treatment prevents transition to persistent hyperalgesia in LysM-GRK2^+/−^ mice

**Figure 4:**
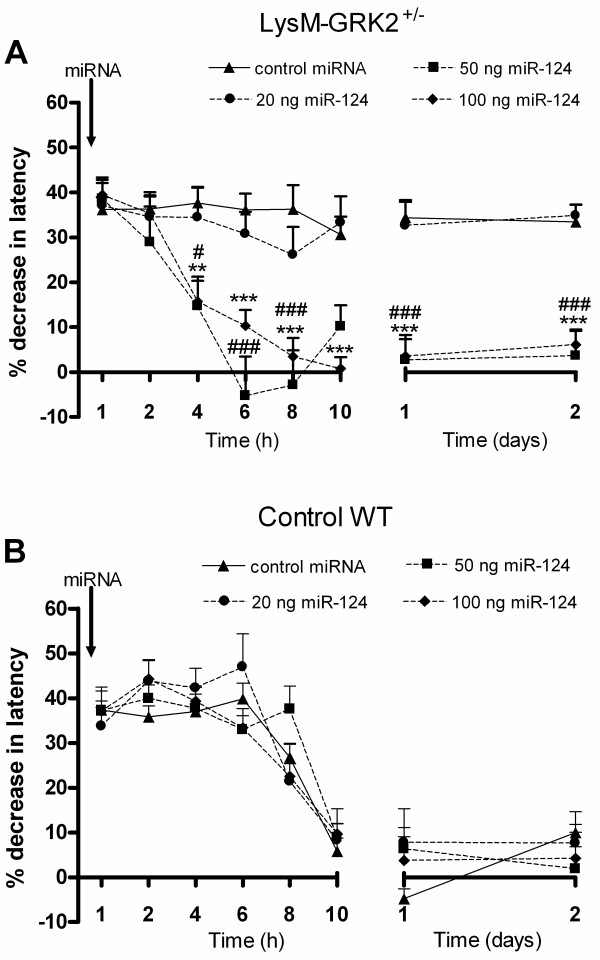
**Effect of microRNA (miR)-124 treatment on interleukin (IL)-1β-induced hyperalgesia.** (**A**) LysM-G protein–coupled receptor kinase (GRK)2^+/−^ and (**B**) wild-type (WT) mice received an intrathecal injection of 20, 50 or 100 ng miR-124 per mouse or 100 ng negative control miRNA 1 day before intraplantar injection of 1 ng IL-1β and the percentage change in heat-withdrawal latency was determined (n = 4 to 10 per group). Data are expressed as means ± SEM. **P < 0.01, ***P < 0.001 for 50 ng miR-124 versus control miRNA; #*P* < 0.05, ###*P* < 0.001 for 100 ng miR-124 versus control miRNA.

To examine the contribution of miR-124 to the course of hyperalgesia, we treated WT and LysM-GRK2^+/−^ mice with an intrathecal injection of miR-124, and monitored its effect on intraplantar IL-1β-induced hyperalgesia. Intrathecal administration of 50 ng and 100 ng miR-124 completely prevented the transition from acute to persistent IL-1β-induced hyperalgesia in LysM-GRK2^+/−^ mice (Figure 4A). whereas intrathecal administration of 100 ng negative control miRNA or the lowest dose of miR-124 tested (20 ng) did not have any effect on the course of hyperalgesia in LysM-GRK2^+/−^ mice (Figure 4A). In WT mice, intrathecal administration of 20 to 100 ng miR-124 did not have any effect on IL-1β-induced hyperalgesia (Figure 4B). In addition, baseline thermal sensitivity was not affected by miRNA administration either (decrease in heat withdrawal latency; WT plus control miRNA 2.6 ± 1.9%; WT plus miR-124 1.8 ± 3.7%; LysM-GRK2^+/−^ plus control miRNA 2.0 ± 3.25%; LysM-GRK2^+/−^ plus miR-124 0.7 ± 3.2%; n = 4).

### Role of miR-124 in regulating spinal cord M1/M2 phenotype

**Figure 5:**
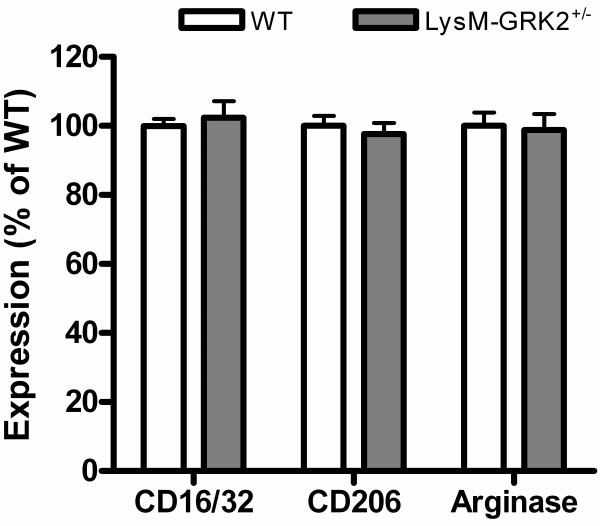
**MicroRNA-124 treatment influences the M1/M2 balance and abolishes the genotype differences after interleukin (IL)-1βinjection.** Mice received an intrathecal injection of 100 ng miR-124 the day before intraplantar injection of 1 ng IL-1β. At 15 hours after IL-1β injection, the spinal cord was collected, and frozen sections of lumbar spinal cord (L2 to L5) were stained for M1 and M2 phenotypic markers. M1 and M2 marker expressions were compared in wild-type (WT) versus LysM-G protein–coupled receptor kinase (GRK)2^+/−^ mice, and expression was quantified in 18 dorsal and 18 lumbar spinal-cord sections per group from 4 mice per group. The level of expression in IL-1β-treated WT mice was set at 100%. Data are expressed as means ± SEM.

After intraplantar IL-1β injection, expression of M1 marker was increased and the expression of M2 marker was significantly decreased in LysM-GRK2^+/−^ mice compared with WT mice (Figure 2). We hypothesized that miR-124 treatment would normalize the expression of the M1 and M2 markers in LysM-GRK2^+/−^ mice. Indeed, the data clearly showed that miR-124 treatment regulates the M1/M2 balance; the intrathecal miR-124 reversed the difference in the expression of M1 and M2 phenotypic markers between WT and LysM-GRK2^+/−^ mice in response to intraplantar IL-1β injection (Figure 5).

### Effect of microRNA-124 on carrageenan-induced inflammatory hyperalgesia in wild-type mice

**Figure 6:**
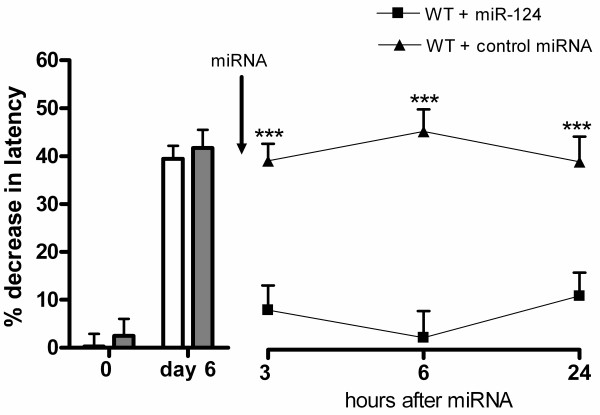
**Effect of microRNA(miR)-124 treatment on carrageenan-induced persistent hyperalgesia in wild-type (WT) mice.** Wild-type (WT) mice received an intraplantar injection of 20 μl 2% λ-carrageenan. At day 6, mice were still hyperalgesic. At this time point, mice received an intrathecal injection of 100 ng miR-124 or control miRNA, and the percentage change in heat withdrawal latency was determined (miR-124 n = 8; control miRNA n = 4). Data are expressed as means ± SEM. ***P < 0.001.

The next question we addressed is whether intrathecal miR-124 treatment can also reverse the persistent hyperalgesia that develops in WT mice after intraplantar injection of carrageenan [8,9,27,28]. WT mice were still hyperalgesic 6 days after an intraplantar injection of high-dose λ-carrageenan. Intrathecal treatment with miR-124 at day 6 rapidly attenuated this persistent carrageenan-induced thermal hyperalgesia (Figure 6).

### Effect of microRNA-124 in a model of neuropathic pain

**Figure 7:**
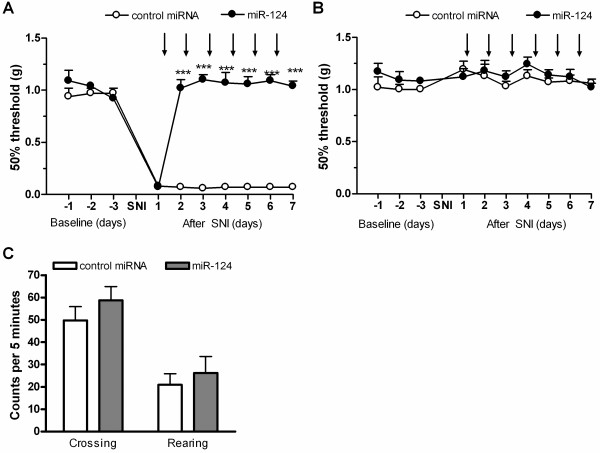
**Effect of miR-124 treatment on SNI-induced mechanical allodynia in WT mice.** Spared nerve injury was performed on WT mice and mice were treated daily with 100 ng miR-124 or control miRNA intrathecally (n = 7 per group). Mechanical allodynia was monitored in (**A**) ipsi- and (**B**) contralateral paw and is depicted as force needed for 50% withdrawal determined in accordance with the up and down method. Arrows indicate miRNA injection. (**C**) Spontaneous locomotor activity in an open field was monitored at day three after SNI to control for a potential effect of miR-124 treatment on motor function. Data are expressed as mean ± SEM. *** *P* < 0.01.

We used the SNI model of chronic neuropathic pain in WT mice to investigate the effect of intrathecal miR-124 treatment on mechanical allodynia as assessed by von Frey filaments. The miR-124 or control miRNA was injected intrathecally once every day, starting 1 day after SNI surgery. Mice treated with control miRNA developed unilateral mechanical allodynia (Figure 7A). The miR-124 treatment completely prevented the mechanical allodynia that develops in the ipsilateral paw in response to SNI (Figure 7A), but did not affect mechanical sensitivity in the contralateral paw (Figure 7B). The miR-124 treatment did not affect spontaneous locomotor activity of SNI mice as determined in an open field 3 days after SNI (Figure 7C).

## Discussion

In this paper, we present a previously unreported role of miR-124 in the regulation of chronic inflammatory and neuropathic pain. We found that intraplantar IL-1β administration to LysM-GRK2^+/−^ mice, which induces persistent hyperalgesia, decreased the level of miR-124 in spinal cord microglia, increased expression of M1 type phenotypic markers and pro-inflammatory cytokines, and decreased expression of anti-inflammatory cytokines and M2 markers.We also showed that intrathecal administration of miR-124 normalized the M1:M2 ratio and prevented transition to persistent IL-1β-induced hyperalgesia in GRK2-deficient mice. Moreover, this is the first study, to our knowledge, to show that intrathecal miR-124 treatment reverses persistent carrageenan-induced hyperalgesia in WT mice. Finally, we found that miR-124 treatment completely inhibits mechanical allodynia in the SNI model of chronic neuropathic pain. Collectively, our findings indicate for that miR-124 could represent a novel treatment for chronic pain.

We showed previously that chronic carrageenan-induced paw inflammation in WT mice is associated with a decrease in GRK2 in spinal cord microglia [9]. In addition, in the spinal nerve transection model of chronic neuropathic pain in rats, spinal-cord microglial GRK2 levels are also reduced by approximately 50% [8]. In the current study, we found that intraplantar IL-1β injection in mice with low microglial/macrophage GRK2 levels induced a reduction in miR-124 expression, leading to a change in the M1/M2 balance towards a more pro-inflammatory phenotype and persistent hyperalgesia. Collectively, our current and previous findings indicate that the mechanisms involved in transition from acute to persistent hyperalgesia in the heterozygous GRK2 knockout model may well be applicable to the chronic pain that develops in more classic models of chronic pain. Indeed, our present findings show that miR-124 treatment also abrogates the existing persistent hyperalgesia induced by carrageenan in WT mice. The clinical relevance is further supported by the current data showing that miR-124 treatment inhibited the development of hyperalgesia in the SNI model of neuropathic pain supports.

It has been shown recently that intracranial injection of a miR-124 antisense oligonucleotide inhibitor induced activation of cerebral microglia [1]. In addition, *in vitro* bone marrow-derived macrophages transfected with miR-124 produced reduced levels of iNOS and increased levels of TGF-β in conjunction with decreased C/EBP-α expression. The current study is the first, to our knowledge, to show that miR-124 treatment prevents transition to persistent hyperalgesia in mice with low GRK2 levels in microglia/macrophages. We also found that treatment with miR-124 reversed the increased M1:M2 ratio that occurred in response to intraplantar IL-1β in the spinal cord of LysM-GRK2^+/−^ compared with WT mice. At the functional level, we showed that the decrease in miR-124 in LysM-GRK2^+/−^ spinal cord microglia in response to intraplantar IL-1β was associated with increased expression of the pro-inflammatory cytokine IL-1β and the pro-inflammatory enzyme iNOS, and with decreased expression of the anti-inflammatory cytokine TGF-β in spinal-cord microglia. At the same time, C/EBP-α, a ‘master’ transcription factor regulated by miR-124, was increased in spinal cord microglia from LysM-GRK2^+/−^ mice after intraplantar injection of IL-1β. Collectively, our findings support the notion that the decreased miR-124 expression seen in conditions of low GRK2 promotes spinal cord microglial/macrophage activation, leading to an increased M1:M2 ratio and prolonged inflammatory hyperalgesia.

The current *in vivo* data are in line with the results from *in vitro* studies reported by Ponomarev *et al*, who found that *in vitro* miR-124 treatment reduces expression of iNOS and increases TGF-β, arginase I and Fizz expression by activated bone marrow-derived macrophages [1]. Therefore, we propose that miR-124 treatment reverses hyperalgesia by restoring the ratio of M1:M2 microglia/macrophages.

To our knowledge, this is the first study to describe a beneficial effect of miR-124 treatment on inflammatory pain and a decrease in spinal cord microglial miR-124 in a model of persistent hyperalgesia. A few recent studies investigated miR-124 expression in other pain models. Bai *et al*. showed that miR-124 levels were significantly reduced in the trigeminal ganglion in a model of carrageenan-induced muscle pain, indicating that inflammatory activity regulates miR-124 in multiple pain models [29]. Brandenburger *et al*. reported that total spinal cord miR-124 was not changed in the chronic constriction injury model of neuropathic pain [30]; however, because miR-124 is expressed at a high level in neurons, a potential change in microglial miR-124 may well have been masked in that study. In the present study, by contrast, miR-124 levels were quantified in isolated spinal microglia.

Because we injected miR-124 intrathecally, we cannot completely exclude that miR-124 also directly affects other cells in the spinal cord, including sensory neurons. Notably, however, in control WT mice we did not observe any effect of miR-124 treatment on the magnitude or duration of IL-1β-induced hyperalgesia. Moreover, intrathecal administration of miR-124 did not affect thermal sensitivity at baseline or mechanical sensitivity in the contralateral paw in the SNI model and did not affect spontaneous locomotor activity. A study using a conditional Dicer knockout mice found that nociceptor miRNA transcripts are required for lowering peripheral pain thresholds in inflammatory hyperalgesia; nociceptor specific deletion of Dicer, an enzyme required for generation of miRNAs, prevents the development of inflammatory hyperalgesia [31]. Based on these and our present findings it is unlikely that the inhibition of persistent hyperalgesia by miR-124 treatment in our study is mediated by inhibition of neuronal hypersensitivity or by impaired motor function.

Intraplantar IL-1β administration did not have any effect on miR-124 expression in spinal cord microglia from control WT mice, whereas it significantly reduced miR-124 levels in microglia from LysM-GRK2^+/−^ mice. This finding indicates that low GRK2 in microglia/macrophages sensitizes these cells for an intraplantar IL-1β-induced decrease in miR-124 expression in spinal cord microglia. The question arises how intraplantar administration of IL-1β induces a decrease in microglial miR-124 levels. Interestingly, *in vitro* it has been shown that co-culture of bone marrow derived macrophages with primary neurons reduces the level of macrophage miR-124 [1]. These findings indicate that neuronal signals can directly regulate the expression of miR-124 in macrophage- like cells. It is known that peripheral nociceptors express IL-1β receptors and triggering of these receptors results in direct sensitisation of the nociceptor [32]. Therefore, it is conceivable that concomitantly, neuronal signals transmitted to the spinal cord induce a reduction in miR-124 and thereby induce a M1 type spinal cord microglia activation. Our finding that only in mice with low GRK2 in microglia/macrophages miR-124 levels in spinal cord microglia are reduced in response to intraplantar IL-1β may point to a contribution of a GPCR-mediated signal to nociceptor-to-microglia signaling. However, GRK2 is also known to regulate cellular signaling at various levels downstream of the membrane receptors. Thus, also increased signaling independently of GPCRs in GRK2-deficient spinal cord microglia/macrophages may contribute to the seen decrease in miR-124 expression in microglia/macrophages from LysM-GRK2^+/−^ mice after intraplantar IL-1β.

## Conclusions

We propose that low spinal microglia/macrophage GRK2 levels promote a transition to persistent hyperalgesia via impaired microglial miR-124 regulation, and consequently an impaired spinal M1/M2 balance. This model is supported by our findings that treatment with intrathecal administration of miR-124 normalized the expression of M1 and M2 markers in LysM-GRK2^+/−^ mice and inhibited the development of IL-1β-induced persistent hyperalgesia in these mice. The broader relevance of our results is supported by our finding that miR-124 treatment reversed persistent hyperalgesia induced by a high dose of carrageenan and prevented development of hyperalgesia in the SNI model of chronic neuropathic pain. In conclusion, miR-124 might represent a novel option for the treatment of chronic pain.

## Competing interests

The authors declare that they have no competing interests.

## Authors’ contributions

HLDM designed and performed experiments, and drafted the manuscript. XJH and QLML performed experiments and revised the manuscript. JZ performed immunohistochemistry and data analysis. CJH and AK designed experiments, supervised data analysis, and revised the manuscript. All authors have read and approved the final manuscript.
